# Efficacy of extracellular vesicles from dental pulp stem cells for bone regeneration in rat calvarial bone defects

**DOI:** 10.1186/s41232-021-00163-w

**Published:** 2021-04-14

**Authors:** Yuka Imanishi, Masaki Hata, Ryohei Matsukawa, Atsushi Aoyagi, Maiko Omi, Makoto Mizutani, Keiko Naruse, Shogo Ozawa, Masaki Honda, Tatsuaki Matsubara, Jun Takebe

**Affiliations:** 1 Department of Removable Prosthodontics, School of Dentistry, Aichi Gakuin University, 2-11 Suemori-dori, Chikusa-ku, Nagoya, 464-8651 Japan; 2 Department of Oral Anatomy, School of Dentistry, Aichi Gakuin University, Nagoya, Japan; 3 Department of Internal Medicine, School of Dentistry, Aichi Gakuin University, Nagoya, Japan

**Keywords:** Dental pulp stem cells, Extracellular vesicles, Regenerative medicine, Bone defects

## Abstract

**Background:**

Extracellular vesicles (EVs) are known to be secreted by various cells. In particular, mesenchymal stem cell (MSC)-derived EVs (MSC-EVs) have tissue repair capacity and anti-inflammatory properties. Dental pulp stem cells (DPSCs), which are MSCs isolated from pulp tissue, are less invasive to the body than other MSCs and can be collected from young individuals. In this study, we investigated the efficacy of EVs secreted by DPSCs (DPSC-EVs) for bone formation.

**Methods:**

DPSC-EVs were isolated from the cell culture medium of DPSCs. DPSC-EVs were unilaterally injected along with collagen (COL), beta-tricalcium phosphate (β-TCP) or hydroxyapatite (HA) into rat calvarial bone defects. The effects of DPSC-EVs were analyzed by micro-computed tomography (micro-CT) and histological observation.

**Results:**

Micro-CT showed that administration of DPSC-EVs with the abovementioned scaffolds resulted in bone formation in the periphery of the defects. DPSC-EVs/COL specifically resulted in bone formation in the center of the defects. Histological observation revealed that DPSC-EVs/COL promoted new bone formation. Administration of DPSC-EVs/COL had almost the same effect on the bone defect site as transplantation of DPSCs/COL.

**Conclusions:**

These results suggest that DPSC-EVs may be effective tools for bone tissue regeneration.

## Introduction

Regenerative therapy with stem cells has the potential to treat intractable diseases and serious tissue damage. Among the cells types thought to be therapeutically efficacious, mesenchymal stem cells (MSCs) are tissue stem cells and are present in adults. MSCs are associated with fewer ethical problems than embryonic stem (ES) cells and have a lower risk of tumorigenesis than induced pluripotent stem cells (iPSCs) [1]. MSCs have the ability to differentiate into multiple cell lineages; colonize, proliferate, and differentiate locally upon transplantation; and alleviate tissue dysfunction by forming new tissues. MSCs can be collected from various tissues, such as bone marrow, fat, the umbilical cord, and dental pulp [2]. Invasive procedures are required to obtain MSCs from bone marrow and fat but not from birth-related tissues such as the placenta, umbilical cord, amniotic fluid, and amniotic membrane.

Dental pulp stem cells (DPSCs) can be collected from third molar and premolars extracted during orthodontic treatment. Therefore, DPSCs are less invasive than other MSCs and can be collected from relatively young individuals. They are thought to be a cell source for regenerative medicine [3, 4].

One advantage of MSCs is that they can be used in the treatment of various diseases, as they show immunomodulatory effects and tissue repair ability [5, 6]. In addition, it is believed that they can be transplanted into allogeneic hosts due to their immunomodulatory effect [7]. Many studies have reported that MSCs are effective in tissue regeneration, but there are still some important issues that need to be addressed, the most important of which is safety [8]. MSCs can be subdivided into different populations based primarily on cell surface phenotype and the ability to differentiate into multiple cell lineages, including osteoclasts, chondrocytes, fat cells, and skeletal muscle cells. There may be heterogeneity within and between MSC populations due to differences in the cell source, such as the sex of the donor, the environmental conditions during isolation, and the tissue from which the cells are derived [9, 10].

The most common method for administering MSCs is intravenous infusion. This route of administration is associated with microvascular occlusion, as cells can become trapped primarily in the capillaries of the lungs before they reach their target site [11]. MSCs have been reported to have the potential to promote fibrosis, which may be another obstacle to their therapeutic use [12, 13]. Given these issues related to MSCs, longer-term studies on the safety of MSC administration may be needed.

New approaches to stem cell therapy are expanding to the use of extracellular vesicles (EVs), which can be used in place of MSCs. EVs are membrane-bound vesicles secreted from cells that include as either exosomes or plasma membrane-derived microvesicles. Exosome, which are vesicles with a lipid bilayer structure, are approximately 30 to 150 nm in diameter and contain genetic substances such as microRNAs and mRNAs. EVs secreted from various cells also participate in cell-to-cell communication [14–17]. Advances in research on tissue regeneration mechanisms have revealed that the release of exosomes by MSCs mediates cell-cell communication and that these exosome supply many components, such as mRNA, DNA, and proteins, to target sites, contributing to tissue repair [18]. Unlike live cells, EVs can be transported and stored for a long period of time, and they do not replicate after being injected. Therefore, EV-based treatments are associated with a lower risk of tumor development and viral pathogen migration than cell-based treatments.

MSC-derived EVs (MSC-EVs) are thought to be as safe and immunologically acceptable as hypoimmunogenic MSCs, as they are essentially trace components of MSCs. MSC-EVs have been shown to exert a regulatory effect on the immune response [19]. Recent studies have focused on the effects of EVs derived from bone marrow-derived MSCs (BMMSC-EVs) and EVs derived from DPSCs (DPSC-EVs) [20, 21]. However, a thorough understanding of the mechanism of action of EVs is still needed. The appropriate concentration and number of EVs that need to be administered are unclear. Although there are still many unanswered questions, MSC-EVs have been reported to exert therapeutic effects in various disease models and are thought to be efficacious for many diseases. In this study, we examined the effectiveness of bone tissue regeneration therapy using DPSC-EVs.

## Materials and methods

### Preparation of DPSCs

DPSCs were isolated from dental pulp tissue by collecting the incisors of 6-week-old male Sprague-Dawley rats (Chubu Kagakushizai, Nagoya, Japan) and Green fluorescent protein (GFP)-transgenic SD rats (SD-Tg[CAG-EGFP] Cz-0040sb; Japan SLC Inc., Hamamatsu, Japan) using 0.1% collagenase and 0.25% trypsin. DPSCs were cultured in minimum essential medium Eagle, alpha modification (MEM-α; Gibco Laboratories Inc., Grand Island, NY) supplemented with 20% fetal bovine serum (FBS; GIBCO) and 1% penicillin streptomycin at 37 °C in a humidified 5% CO_2_ incubator. Nonadherent cells were washed away, and adherent cells were grown continuously until passage 3. DPSCs were used at passages 3–6 for all experiments.

This study was approved by the Institutional Animal Care and Use Committee of Aichi Gakuin University (AGUD 437-2), and all animal experiments were carried out following national guidelines and the relevant national laws on the protection of animals.

### Flow cytometry

DPSCs were characterized using fluorescence-activated cell sorting (FACS; MACSQunat analyzer; Miltenyi Biotec, Bergisch Gladbach, Germany). Cells were stained with FITC-conjugated mouse monoclonal antibodies against rat CD49d and rat CD90 (Becton Dickinson, Franklin Lakes, NJ), a FITC-conjugated hamster antibody against rat CD29 (Becton Dickinson) and PE-conjugated mouse monoclonal antibodies against rat CD34 and rat CD45 (Becton Dickinson). Antibodies of the same isotype were used as controls. The data were analyzed with MACS Quantify software (Miltenyi Biotec).

### Cell proliferation assay

The proliferative capacity of DPSCs was assessed with Cell Counting kit-8 (CCK-8) according to the manufacturer’s procedure (DOJINDO LABORATORIES, Kumamoto, Japan). DPSCs were seed onto 24-well plates at 3 × 10^4^ cells/well. After 3, 5, and 7 days of culture, CCK-8 regent was added and then cells were incubated for 3 h at 37 °C in a humidified 5% CO_2_ incubator. The absorbance was determined at 450 nm with a microplate reader (SPARK 10 M; Tecan Japan Co., Ltd., Kanagawa, Japan).

### Analysis of adipogenic, osteogenic, and chondrogenic differentiation by staining

DPSCs were differentiated into adipocytes, osteoblasts, or chondrocytes using adipogenic osteogenic or chondrogenic differentiation-inducing medium (Lonza, Basel, Switzerland) according to the manufacturer’s instructions. Cells were stained with Oil red O (Polysciences, Warrington, PA) and fatty acid-binding protein-4 (FABP-4; R & D Systems, Minneapolis, MN) to assess adipogenic differentiation. Cells were stained with alkaline phosphatase (ALP; Millipore, Billerica, MA) and osteocalcin (R & D Systems) to assess osteogenic differentiation. Cells were stained with aggrecan (R & D Systems) to assess chondrogenic differentiation. For the detection of nuclei, cells were stained with 4′-6-Diamidino-2-phenylindole (DAPI; Sigma Aldrich, St. Louis, MO).

### Isolation and identification of EVs

DPSCs (1 × 10^5^/well) were seeded in a six-well plate. After the cells were cultured in MEM-α medium supplemented with 20% FBS and 1% penicillin streptomycin for 24 h, the medium was changed to serum-free medium, and the cells were cultured for 48 h [22]. The serum-free medium was collected and centrifuged at 2000×*g* to remove the precipitated cell components. DPSC-EVs were extracted from the obtained culture supernatant using Total Exosome Isolation Reagent (Thermo Fisher Scientific, Waltham, MA). According to the manufacturer’s protocol, a 0.5 volume of Total Exosome Isolation Reagent was added to the culture supernatant, and the mixture was allowed to stand at 4 °C overnight and then centrifuged at 2 °C and 10,000×*g* for 1 h. The DPSC-EV pellets were resuspended in phosphate-buffered saline (PBS) and stored at − 30 °C.

### Transmission electron microscopy

The morphology of EVs was examined by transmission electron microscopy (TEM). EVs in 2 μl PBS were placed on a hydrophilized Excel 200 mesh Cu support film (Nissin EM, Tokyo, Japan) and incubated at room temperature for 2 min. The Excel support film was treated with 10 μl uranyl acetate for 10 s, completely dried, subjected to carbon vapor deposition (IB-3 ION Coater) (Eiko Engineering, Ibaraki, Japan), and observed by TEM.

### Western blot analysis

Samples containing EVs were electrophoresed on a polyacrylamide gel and transferred to a polyvinylidene difluoride membrane (Bio-Rad, Hercules, CA). The membrane was incubated at 4 °C with antibodies against CD9 (Abcam, Cambridge, UK) and α-tubulin (Cell Signaling Technology, Danvers, MA) followed by an HRP-conjugated anti-rabbit monoclonal IgG antibody (System Biosciences, Palo Alto, CA). The proteins were visualized using an ECL plus chemiluminescence detection kit (Thermo Fisher Scientific).

### Animals and surgical procedure

**Fig 1.**
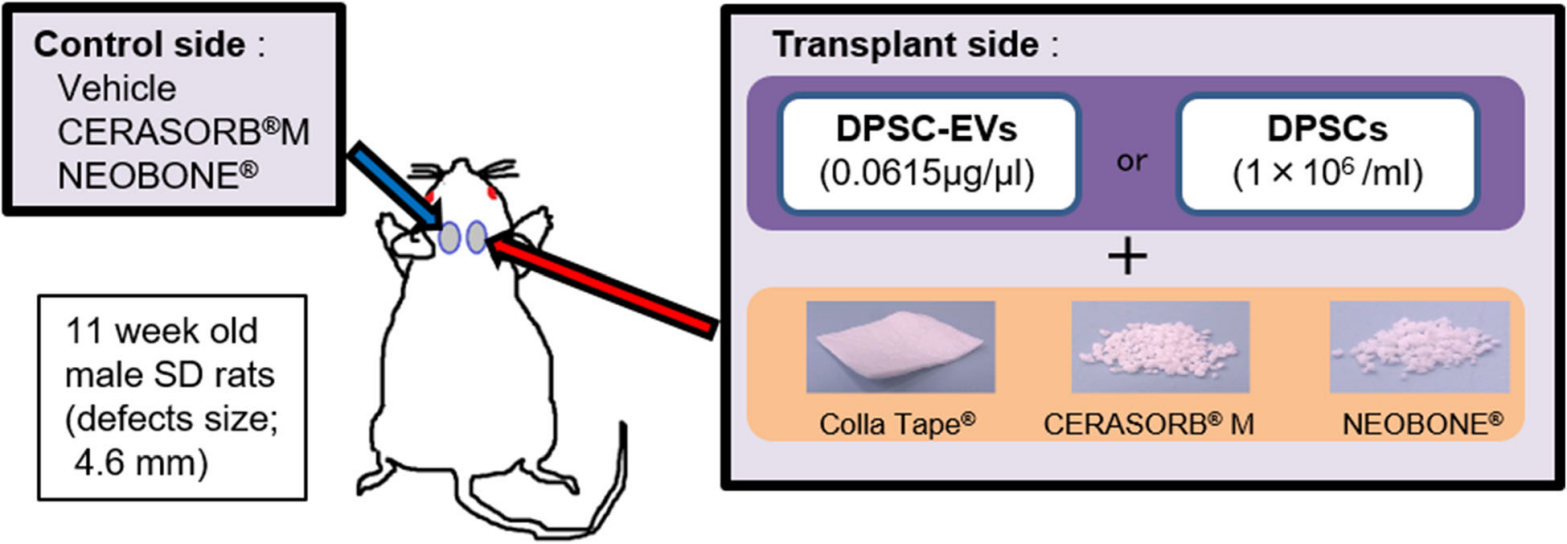
Experimental protocol. Defects with a diameter of 4.6 mm were created in the parietal bones of 11-week-old SD rats. DPSCs or DPSC-EVs and scaffold materials were transplanted into the defect on the right side. Vehicle was administered and scaffold materials were filled to the control defect on the left side

Eleven-week-old male SD rats were anesthetized, and dental trephine bars were used to create 4.6-mm-diameter critical-sized defects on both sides of the rat calvarial bone (Fig. 1).

One of three materials, namely collagen (COL; Colla Tape®, Integra Life Sciences, Princeton, NJ), β-tricalcium phosphate (β-TCP; CERASORB® M, Curasan AG, Kleinostheim, Germany), or hydroxyapatite (HA; NEOBONE®, CoorsTek KK, Tokyo, Japan), was transplanted into the calvarial bone defect on the right side as a scaffold, and DPSCs (1 × 10^6^/ml) or DPSC-EVs (0.0615 μg/μl) were injected. The left defect was filled with β-TCP or HA or injected with vehicle as a control.

### Micro-computed tomography

The calvarial bones were observed under anesthesia using micro-computed tomography (micro-CT) (COSMO Scan GX, Rigaku, Tokyo, Japan) 4 weeks after the surgical procedure [23]. Micro-CT was performed after 18 s of exposure. The tube voltage was 90 kV, the tube current was 88 μA, and the image size was 512 × 512 pixels. Three-dimensional images, which contained horizontal sections and sagittal sections, were reconstructed and volumes of newly formed bone areas were calculated using TRI/3DBONE software (RATOC System Engineering, Tokyo, Japan).

### Histological analysis

The calvarial bones were harvested 16 weeks after the surgical procedure and fixed with 10% formalin solution (Muto Pure Chemicals Co., Tokyo, Japan). Frozen sections of the samples were prepared by the adhesive film method [24]. The samples were decalcified using 20% Plank-Rychlo solution (Muto Pure Chemicals Co.) for 4 days at room temperature, neutralized with 5% sodium sulfate solution, and washed with running water for 24 h. The samples were cryoembedded in super cryoembedding medium (SCEM) gel in isopentane cooled in liquid nitrogen. The frozen SCEM block was cut into 5-μm-thick continuous sections with a tungsten carbide razor blade (TC-65, Leica Microsystems, Wetzlar, Germany) using a microtome (CM3050S, Leica Microsystems) after the adhesive film was attached to the cutting surface. The sections were stained with hematoxylin-eosin and analyzed using a light microscope. Osteocalcin was detected by immunohistochemical staining. The sections were incubated overnight at 4 °C with primary antibody (anti-Osteocalcin antibody; R&D) diluted 1:50 and subsequently stained using the Histofine Simplestain rat system (Nichirei., Tokyo, Japan) according to the manufacturer’s instructions and observed using a light microscope.

### Statistical analysis

Results were expressed as means ± standard error of the mean (SEM). Statistical analyses were performed by one-way ANOVA with Bonferroni correction for multiple comparisons. The significant difference was indicated at the *P* < 0.05.

## Results

### Identification of DPSCs and DPSC-EVs

**Fig. 2:**
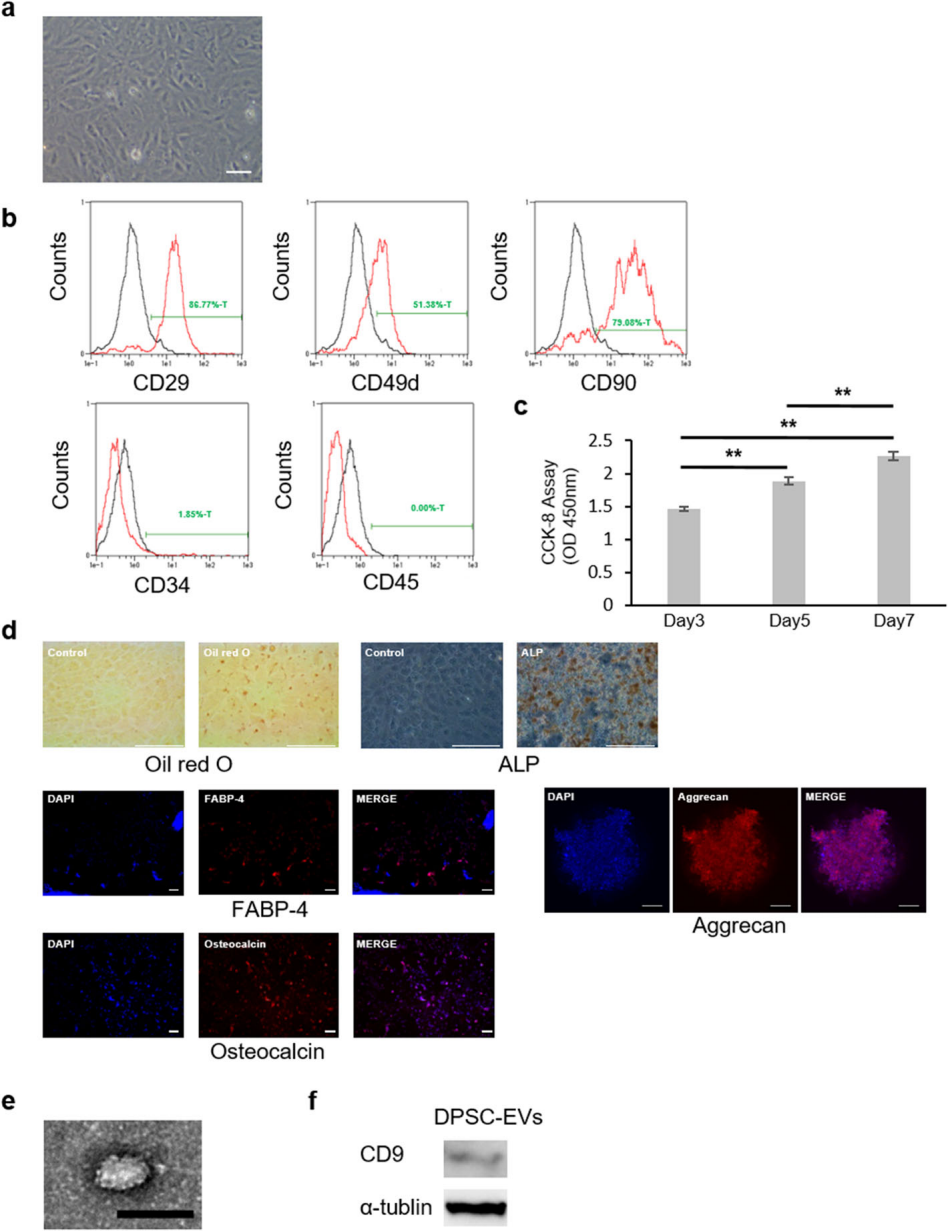
Characterization of DPSCs and DPSC-EVs. **a** Cultured DPSCs observed under a phase contrast microscope. Bars = 100 μm. **b** Flow cytometric histograms of DPSCs. The expression of the cell surface markers CD29, CD34, CD45, CD49d, and CD90 was analyzed. Black line: isotype control. Red line: stained with specific antibodies. **c** The proliferation of DPSCs was assessed by the CCK-8 assay on day 3, day 5, and day 7. Results are expressed as means ± SEM (*n* = 8). **d** The capacity of DPSCs to differentiate into adipocytes, osteoblasts and chondrocytes was assessed by Oil red O, ALP, FABP-4, osteocalcin and aggrecan staining. Bars = 100 μm. **e** DPSC-EV morphology observed using TEM. Bars = 100 nm. **f** Western blot analysis of the expression of the EV surface marker CD9 in DPSC-EVs

Cultured DPSCs exhibited a typical spindle-shaped morphology, as visualized by phase-contrast microscopy (Fig. 2a). Flow cytometric analyses showed that DPSCs were positive for CD29, CD49d, and CD90 and negative for CD34 and CD45. Expression rates of surface antigen on cytoplasmic membrane were CD29 (89.0 ± 2.18%), CD49d (60.3 ± 5.81%), CD90 (82.9 ± 1.82%), CD34 (1.72 ± 0.49%), CD45 (0.06 ± 0.04%) (*n* = 3) (Fig. 2b). Cell proliferation analysis showed that proliferation of DPSCs were significantly increased on day 3, day 5, and day 7 (*P* < 0.01) (Fig. 2c). Isolated DPSCs exhibited Oil red O and ALP staining and were positive for FABP-4, osteocalcin, and aggrecan under adipogenic, osteogenic, and chondrogenic culture conditions. These data indicated that isolated DPSCs possessed the ability to differentiate into adipocytes, osteoblasts and chondrocytes (Fig. 2d).

EVs were purified from DPSCs-conditioned cultured medium based on the manufacturer’s protocol, and TEM revealed that the EVs exhibited a circular morphology and a diameter of 100 nm (Fig. 2e). The expression of specific surface markers was measured with a CD9 antibody by Western blot analysis (Fig. 2f).

### Efficacy of DPSC-EVs as tools for bone regeneration

**Fig. 3:**
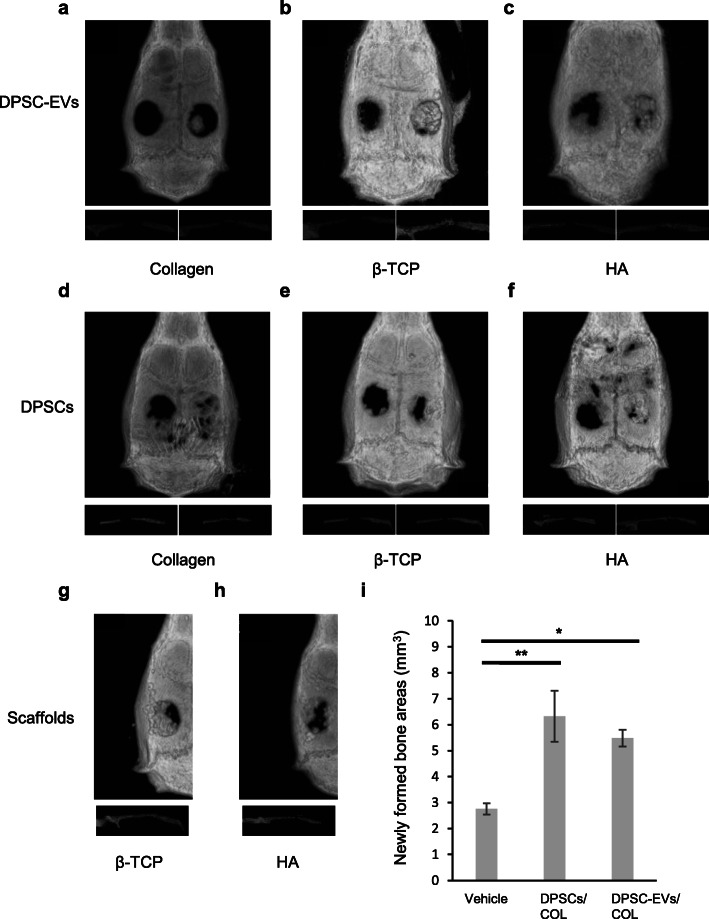
Micro-CT analysis of calvarial defects 4 weeks after the procedure. Administration of DPSC-EVs with COL (**a**), β-TCP (**b**), and HA (**c**). Transplantation of DPSCs with COL (**d**), β-TCP (**e**), and HA (**f**). Filling of β-TCP (**g**) and HA (**h**). The upper image is a horizontal section. The lower images are left and right sagittal sections. **i** Volume of newly formed bone areas was measured by 3D images. Results are expressed as means ± SEM (*n* = 6)

**Fig. 4:**
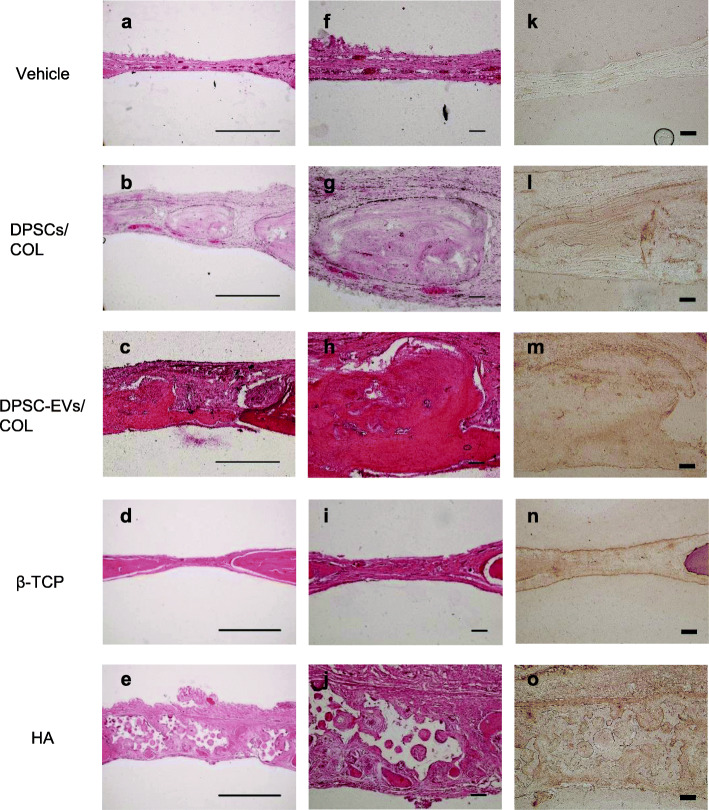
Histological analysis of the calvarial defect 16 weeks after the procedure. Sagittal sections of parietal bone were stained with hematoxylin-eosin and immunohistochemical staining for osteocalcin. **a**, **f**, **k** Vehicle. **b**, **g**, **l** DPSCs/COL. **c**, **h**, **m** DPSC-EVs/COL. **d**, **i**, **n** β-TCP. **e**, **j**, **o** HA. Left section: × 4 magnification, bar = 1 mm. Middle and Right section: × 10 magnification, bar = 0.1 mm

To assess bone regeneration induced by DPSC-EVs at the calvarial bone defect, micro-CT was performed 4 weeks after the surgical procedure. Hard tissue was found in the center of the defect in horizontal and sagittal sections from both the DPSC-EVs/COL groups and the DPSCs/COL groups (Fig. 3a, d). In the DPSC-EVs/β-TCP group and the DPSC-EVs/HA group, the presence of hard granular tissue in the defect was confirmed (Fig. 3b, c). In the DPSCs/β-TCP group and the DPSCs/HA group, the presence of hard bone-like tissue and hard granular tissue in the defect margin was confirmed (Fig. 3e, f). Bone tissue was found in the defect margin on the control side, which was vehicle group. β-TCP group and HA group showed hard granular tissue in the defect (Fig. 3g, h). Quantitative analysis revealed that volumes of newly formed bone areas in the DPSCs/COL group and DPSC-EVs/COL group were significantly increased compared with vehicle group (*P* < 0.05). There were no differences between DPSCs/COL group and DPSC-EVs/COL group (Fig. 3i). Furthermore, histological analysis also showed that newly formed bone was present in the central part and margin of the defect in both the DPSC-EVs/COL group and the DPSCs/COL group 16 weeks after the surgical procedure (Fig. 4b, c, g, h). β-TCP group were observed that β-TCP granules were absorbed and newly formed bone was present in the margin of the defect (Fig. 4d, i). HA group were observed that HA granules and newly formed bone were present in the defect (Fig. 4e, j). The vehicle group exhibited fibrous connective tissue (Fig. 4a, f). Inflammatory responses were not observed in any of the groups. Immunohistochemical staining showed that osteocalcin were detected with positive brown staining on DPSCs/COL group and DPSC-EVs/COL group in the central part of the defect (Fig. 4l, m). β-TCP group and HA group were observed with positive brown staining in the margin of the defect (Fig. 4n, o). There was no detection for osteocalcin on the vehicle group (Fig. 4k).

## Discussion

In this study, we confirmed that the combination of DPSC-EVs and each of the three scaffold materials (COL, β-TCP, and HA) promoted bone tissue formation in critical-sized rat calvarial bone defects. The effects of the combination of DPSC-EVs and different scaffolds were compared using X-ray micro-CT, HE staining, and immunohistochemical staining. We also evaluated the bone tissue-forming ability of DPSC-EVs/COL. Cellular therapy is a major research area in bone regeneration. However, cell-based treatments are closely associated with tumors and embolization [25]. The intravenous administration of cellular paracrine factors rather than cells avoids the risk of embolization and reduces the risk of tumorigenesis due to uncontrolled cell differentiation.

EVs is a comprehensive term for a lipid bilayer vesicle located outside cells and include exosome, microvesicles, and apoptotic bodies [26]. Exosome, which are approximately 100 nm in diameter, are associated with fewer safety concerns, are capable of promoting bone formation, and may be used for new treatments for bone tissue repair [27, 28]. Thus, in recent years, MSC-derived exosome have attracted increasing attention as non-cell factors for tissue repair [29]. DPSCs are attractive cell sources due to their low invasiveness and ability to be collected from young individuals. DPSC-derived exosomes have also been reported to promote bone healing with biodegradable triblock copolymer microsperes [30]. In our study, we confirmed that the combined administration of DPSC-EVs and different scaffold materials (COL, β-TCP, or HA) can repair tissue in critical-sized rat calvarial bone defects. Compared to scaffold materials alone, the combination of MSC-derived-exosome with scaffold materials promotes broader angiogenesis and results in the formation of better bone tissue [28]. To date, COL, β-TCP, and HA have been used as scaffolds in combination with exosome. The combination of exosome and scaffolds exerts a better bone regenerative effect than scaffold materials alone [31–35].

In this study, all DPSC-EVs/scaffold materials promoted new bone formation in the margin of the bone defect. DPSC-EVs/COL formed new bone in the center of the bone defect. In a comparative in vitro study of different materials with rat bone mesenchymal stem cells (rBMSCs), COL/HA composites, COL, biphasic calcium phosphate (BCP), and HA were shown to have good cell compatibility. The study also showed that these materials can promote bone formation and the differentiation of rBMSCs. COL/HA composites COL and BCP were superior to HA in their ability to promote bone formation and the differentiation of rBMSCs [36]. BMSC derived-exosomes directly bind to extracellular matrix proteins such as type I collagen and fibronectin and showed the osteogenic potential in regenerative medicine [37]. Unlike DPSC-EVs/COL, DPSC-EVs/β-TCP promoted bone tissue regeneration while maintaining the thickness of the bone defect. β-TCP binds directly to bone, and its granules rapidly dissolve, filling the area with newly formed bone-dissolved granules [38, 39]. In the future, to select the most suitable scaffold, it will be necessary to compare the effects of transplantation of a scaffold alone with the effects of transplantation of the combination of EVs and the scaffold. In addition, long-term continuous evaluation of the speed and quality of the effect of EVs on promoting the natural healing ability of bone is required.

To determine the effect of DPSC-EVs, it is necessary to evaluate the bone formation effect of both DPSC-EVs/scaffold and DPSCs/scaffold. Observation of the bone defect using X-ray micro-CT showed that 4 weeks after the surgical procedure, DPSC-EVs/scaffold and DPSCs/scaffold promoted the formation of hard tissue in the bone defect. Measurement of newly formed bone volumes was significantly increased in the DPSC-EVs/COL group and the DPSCs/COL group compared with the vehicle group. Histological evaluation using HE staining and immunohistochemical staining with osteocalcin, which is osteogenic marker, revealed osteogenesis in the central part of the defect in the DPSC-EVs/COL group and the DPSCs/COL group 16 weeks after the surgical procedure. In contrast, there was fibrous connective tissue in the vehicle group at the same time point. Although few studies have directly compared the effects of stem cells with those of stem cell-derived EVs, studies comparing the effects of iPSCs and iPSC-derived exosomes on monkey skin wound healing have shown that both autologous and allogeneic iPSCs and exosome derived from these cells maintain the full thickness of rhesus monkey skin. iPSCs and iPSC-derived exosomes have been shown to promote the healing of skin wounds. Additionally, there is no statistically significant difference in wound closure rate following transplantation of iPSCs or iPSC-derived exosomes [40].

Although MSC-EVs have been shown to exert tissue repair effects, the mechanism underlying these effects is not yet fully understood. MSC-EVs have been reported to function as mediators by transporting mRNA and miRNAs, proteins and other molecules to recipient cells, thereby regulating the biological activity of target cells.

Recent studies have suggested that miRNAs carried by exosomes are important for intracellular communication between source and target cells. The ability of MSC-derived exosome to accelerate fracture healing is partially mediated by miRNAs [41]. However, the composition of the exosomes cargo depends on cell origin and the physiological or pathological conditions during the formation of the exosomes [42, 43]. The cargo carried by DPSC-derived exosomes, which may have various effects on bone tissue formation, is also thought to vary depending on the culture conditions. Similarly, an appropriate dose and concentration of exosomes should be selected. Many reports have shown that at higher doses and concentrations, exosomes are relatively efficacious for bone regeneration [34, 35, 44]. However, further studies on the effects of high doses and concentrations of exosomes are needed.

## Conclusions

These results suggest that DPSC-EVs/COL and DPSCs/COL have almost the same effect on the bone defect site and thus are useful for bone tissue regeneration therapy.

## Data Availability

All data generated and/or analyzed during this study are included in this published article. Data sharing is not applicable to this article, as no datasets were generated or analyzed during the current study. However, the data that support the findings of this study are available from the corresponding author upon reasonable request.

## Abbreviations

ALP: Alkaline phosphatase
β-TCP: Beta-tricalcium phosphate
BCP: Biphasic calcium phosphate
BMMSC-EVs: EVs derived from bone marrow-derived MSCs
CCK-8: Cell Counting kit-8
COL: Collagen
DAPI: 4′-6-Diamidino-2-phenylindole
DPSCs: Dental pulp stem cells
DPSC-EVs: EVs derived from DPSCs
ES: Embryonic stem
EVs: Extracellular vesicles
FABP-4: Fatty acid-binding protein-4
FACS: Fluorescence-activated cell sorting
HA: Hydroxyapatite
MEM-α: Minimum essential medium Eagle, alpha modification
micro-CT: Micro-computed tomography
MSCs: Mesenchymal stem cells
MSC-EVs: MSC-derived EVs
iPSCs: Induced pluripotent stem cells
PBS: Phosphate-buffered saline
rBMSCs: Rat bone mesenchymal stem cells
SCEM: Super cryoembedding medium
TEM: Transmission electron microscopy
